# Adaptation of an Emotional Stroop Test for Screening of PTSD Related to Intimate Partner Violence in Spanish-Speaking Women

**DOI:** 10.3390/bs15030343

**Published:** 2025-03-11

**Authors:** Sarai Mata-Gil, Luz M. Fernández-Mateos, Antonio Sánchez-Cabaco, Jerónimo Del Moral-Martínez, Eduardo Castillo-Riedel

**Affiliations:** 1 Faculty of Education and Psychology, Campus Universitario, University of Extremadura (UEX), 06006 Badajoz, Spain; 2 Faculty of Psychology, Pontifical University of Salamanca (UPSA), 37002 Salamanca, Spain; lmfernandezma@upsa.es (L.M.F.-M.); asanchezca@upsa.es (A.S.-C.); 3 Faculty of Science, Campus Universitario, University of Extremadura (UEX), 06006 Badajoz, Spain; jeronimodelmoral@yahoo.es; 4 School of Psychology, CETYS University, Av. Cetys Universidad, nº4, Tijuana 22210, Mexico; eriedel@cetys.edu.mx

**Keywords:** abuse, Emotional Stroop, intimate partner violence (IPV), women, post-traumatic stress disorder (PTSD)

## Abstract

Cognitive assessment instruments with emotional components may be useful to address the limitations of the self-report scales commonly used to assess post-traumatic stress disorder (PTSD) in women victims of domestic violence (IPV). The aim of this study was to develop an Emotional Stroop task designed to identify post-traumatic stress disorder (PTSD) linked to intimate partner violence (IPV) in Spanish-speaking women. The validation of this test involved a comparative analysis between two groups: a clinical group (*n* = 50) and a non-clinical group (*n* = 50) of women with an average age of 38.38 (SD = 12.31; 100% female participants). The study indicates that the clinical group scored significantly higher on the PTSD Symptom Severity Scale (EGS) and lower on the three Stroop tasks compared to the non-clinical group. Notably, there was a significant negative correlation between the results of the modified Stroop tasks and the EGS test. The results show that our adapted Stroop task serves as an efficacious tool for detecting PTSD related to intimate partner violence (IPV) in Spanish-speaking women. Moreover, it has the potential to alleviate the constraints of presently available tools designed for this specific purpose.

## 1. Introduction

Intimate partner violence (IPV) is characterized as any form of abusive behavior involving physical, psychological, and/or sexual mistreatment by one person within an intimate relationship towards another (Labrador et al., 2004), and leads to a significant number of victims worldwide annually. This figure does not account for estimates of unreported or unrecorded incidents (World Health Organization, 2021). It is important to highlight that psychological abuse can have consequences of similar or greater severity compared to those associated with physical abuse (Dokkedahl et al., 2022).

IPV victims maintain a constant state of prolonged vigilance, resulting in exaggerated reactions to neutral stimuli and the perception of a hostile environment. In addition, heightened sensitivity to learned threat behaviors persists, even after leaving the violent situation, causing individuals to continue to live as if the threat persists.

Among women who have experienced abuse, post-traumatic stress disorder (PTSD) stands out as a very significant and prevalent psychological injury (Spencer et al., 2022). PTSD involves a cluster of anxiety symptoms, particularly those related to situations that pose a real threat to one’s life or other threat of physical injury (American Psychiatric Association, 2014).

Several assessment models are used to diagnose PTSD, and while some may not consider the criterion of emotional hypervigilance from a cognitive perspective, focusing instead on increased arousal, the Stroop task inventory is used to assess emotional hypervigilance specifically at the cognitive level (Stroop, 1935). The Stroop task assesses cognitive interference, providing a means to analyze the subject’s capacity to organize information from their surroundings and respond selectively. Through the examination of these two abilities, the Stroop task serves as a specialized tool for measuring dimensions associated with cognitive flexibility, resistance to stimuli interference, creativity, pathology, and cognitive complexity (Golden, 1994).

In the Emotional Stroop task, participants are presented with three sets of stimuli words with positive, negative, or neutral emotional dimensions printed in different colored inks. The emotional content is customized for specific pathologies, and participants must verbally identify the color of each word while disregarding its meaning. For example, they may name the color of the word “blood” printed blue, as opposed to a neutral word like “table” also printed in blue. Throughout this task, participants focus on naming the colors of the words, and these color–word associations vary in relation to distinct psychopathological themes (Williams et al., 1996). Individuals with emotional disorders demonstrate slower verbalization of the color of emotionally charged words, particularly when identifying the color of a word linked to concerns relevant to their clinical state. Attentional bias, a core concept in cognitive theory, underlies many psychopathologies. The Stroop task reveals that those without psychopathology show less pronounced attentional bias compared to individuals with psychopathology. However, studies applying the Stroop task to detect PTSD associated with intimate partner violence (IPV) are scarce (Sippel & Marshall, 2011). Notably, in Spain, the assessment of PTSD related to IPV relies on questionnaires and scales, such as those developed by Echeburúa and collaborators (Echeburúa et al., 1995, 2016, 2017).

Nevertheless, employing questionnaires/scales for assessment has a limitation: individuals can manipulate the test to conceal symptoms, either by refusing to answer truthfully or by aligning responses with the desired expectations of the evaluator or themselves. Such manipulation has the potential to distort the objective outcome. On the other hand, the Emotional Stroop task offers an advantage in that, despite its limitations, voluntary control over the mechanism cannot be carried out, preventing interference and enhancing the reliability of the results (Straub et al., 2022).

Meeting the professional demands of forensic neuropsychology, a substantial portion of the forensic literature is dedicated to probing simulation, specifically the feigning and exaggeration of cognitive symptoms. This circumstance is not an exception for women victims of intimate partner violence (IPV) (Marín Torices et al., 2018).

This study aims to develop a modified version of the Stroop task tailored for detecting post-traumatic stress disorder (PTSD) in Spanish-speaking women victims of intimate partner violence (IVP). This adaptation is designed not only to simplify the evaluation and diagnosis process but also to enhance its effectiveness by minimizing the impact of simulation and pretense. This is particularly relevant given the substantial population of Spanish-speaking women worldwide.

## 2. Materials and Methods

The project was structured in four phases: (1) the acquisition of words for the various sheets, (2) validation of the selected words, (3) implementation of the adapted Emotional Stroop tests for domestic violence victims, and (4) validation of the test through a comparative analysis between the clinical and non-clinical or control samples.

### 2.1. Phase 1—Selection of Words for the Neutral and Positive (or Emotional) Word Sheets

The words corresponding to the sheets of neutral words and positive words were established in previous studies by our work team (Cabaco & Armas, 2000; Cabaco et al., 2002).

### 2.2. Phase 2—Selection and Validation of Words for the Negative (or Activating) Word Sheet

This second phase of the research was subdivided into four stages.

#### 2.2.1. First Stage—Literature Review

For the preparation of the activation sheet, an initial bibliographical search was conducted on studies utilizing the Emotional Stroop paradigm, specifically focusing on words that activate PTSD related to violence against women. A total of 31 words were identified from these sources (see Table 1).

#### 2.2.2. Second Stage—First Selection of Negative (or Activating) Words

A panel of judges was employed comprising of 29 professionals with diverse backgrounds and expertise related to post-traumatic stress disorder (PTSD) and domestic violence situations. This panel included individuals from the National Police Corps, psychologists from victim care centers, directors of Women’s Centers, social workers, and personnel specializing in the field of gender violence.

This expert panel generated a collection of 20 negative words using a fieldwork booklet developed for this purpose. In collaboration with the 31 words identified from the initial literature review, the panel was tasked with classifying the words on a Likert-type scale, assigning scores ranging from 1 to 5 based on their activating capacity (where the score 1 = not at all, 2 = a little, 3 = moderately, 4 = considerably, 5 = very much). Additionally, judges had the option to suggest other relevant words, resulting in an expanded list with approximately 54 proposed words.

The words proposed by the judges were scored based on the number of repetitions in the proposal as negative words.

Based on the results obtained, the initial selection comprised the first 23 negative words (derived from both the literature review and fieldwork), arranged in descending order, with scores ranging from a maximum of 134 to a minimum of 100 points. Additionally, 14 words proposed by the judges, also ranked in descending order of score, were incorporated into the selection. This process resulted in a total of 37 words (see Table 1 and Table 2).

#### 2.2.3. Third Stage—Second Selection of Negative (or Activating) Words

Subsequently, the 37 words underwent an assessment based on four specified formal criteria defined as “Frequency of Use” (indicating how frequently the term is typically employed), “Familiarity” (reflecting the degree of common usage), “Understanding” (evaluating the level at which the term is comprehended through the ability to provide a technical definition), and “Degree of Image Evocation” (measuring the world’s capacity to evoke a mental image).

This assessment was carried out by 97 university subjects enrolled in their first year of Psychology at the Pontifical University of Salamanca, consisting of 79 women (81.44%) and 18 men (18.66%). These individuals have an average sociocultural level and an average age of 19.32 years (SD = 1.23). They represent diverse backgrounds from nine different autonomous communities in Spain.

The assessment was carried out using a Likert-type scale ranging from 0 to 5, where 0 = not at all, 1 = a little, 2 = somewhat, 3 = moderately, 4 = considerably, 5 = very much.

Initially, 10 words were chosen from each formal criterion, arranged in descending order based on the sum of scores. The selection process prioritized the 20 words that consistently accumulated the highest scores across all criteria from the outset.

#### 2.2.4. Fourth Stage—Final Selection of Negative (or Activating) Words

From the initial set of 20 words chosen by university students, the project team ultimately selected 10 words based on various semantic fields, considering the context in which domestic violence and PTSD unfold. These fields include physical violence, verbal violence, sexuality, self-esteem, and the setting of domestic violence. The final selection also considered the prevalence of these words in the scientific press (see Table 3).

### 2.3. Phase 3—Development of the Emotional Stroop Test for Detecting PTSD in Women Affected by Domestic Violence

Following the structure of the Stroop task, each sheet was printed in A4 format (21 × 30 cm). Every sheet comprised 100 words arranged in five columns of 20. To ensure randomness, no word was repeated consecutively within the same column or row, and the same principle applied to the colors. The words constituting the three sheets of the Emotional Stroop Test for detecting PTSD in women victims of domestic violence are detailed in Table 4.

**Figure 1:**
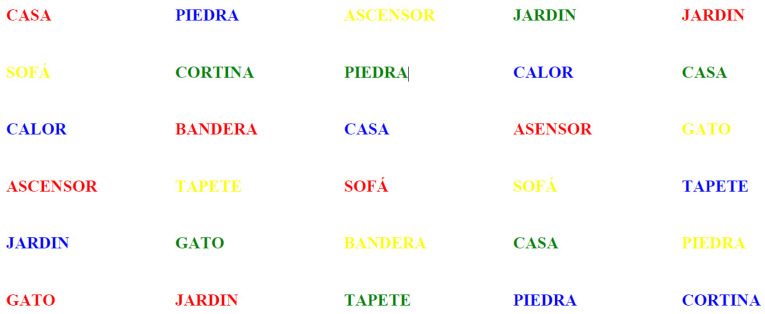
The ten neutral words.

**Figure 2:**
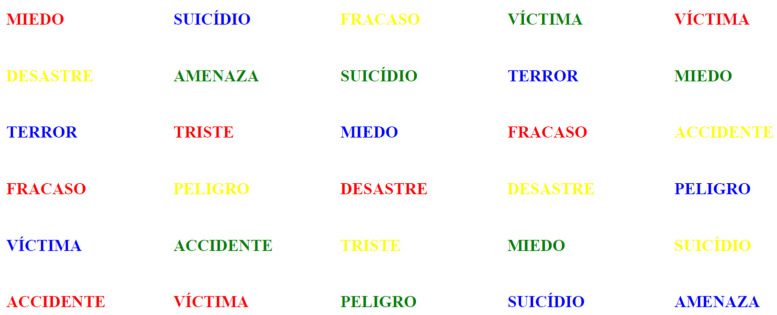
The ten positive words.

**Figure 3:**
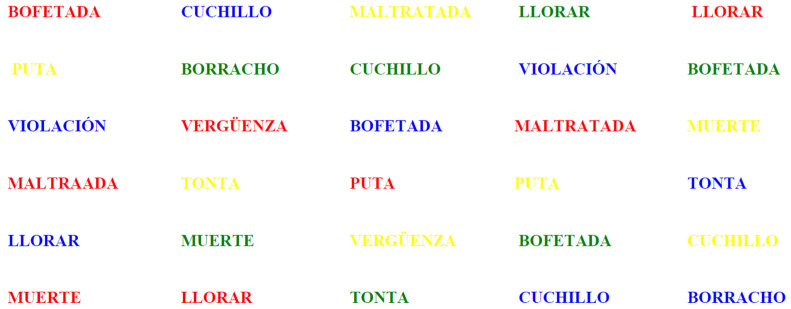
The ten negative words.

Figure 1, Figure 2 and Figure 3 correspond to excerpts from the Emotional Stroop Test for the identification of PTSD in women victims of intimate partner violence (IPV). The initial sheet showcases ten neutral words printed in blue, yellow, green, and red ink (Figure 1).

The second sheet included the ten words classified as positive (or emotional)—words that evoke an emotional response—also printed in color (Figure 2).

The third sheet included the words classified as negative (or activating), also printed in color (Figure 3).

### 2.4. Phase 4—The Final Study: The Emotional Stroop Test for Detecting PTSD in Women Victims of Domestic Violence and the Post-Traumatic Stress Disorder Symptom Severity Scale

For this phase of the study, 100 participants, all female, were involved and divided into two groups: the clinical sample (*n* = 50) consisted of women who were victims of domestic violence, located in shelters (33%) and in the “Unit of Prevention, Assistance, and Protection” (Unidad de Prevención, Asistencia y Protección, UPAP) of abused victims at the “Higher Police Headquarters of Extremadura” (Jefatura Superior de Policías de Extremadura) (17%), and the non-clinical sample (*n* = 50), composed of women who were not victims of domestic violence, including volunteers from the “Association of Centers for Culture and Promotion of Women” (Asociación de Centros de Cultura y Promoción de la Mujer) (34%) or those unaffiliated with any official reference body (16%). All participants from both groups were selected from the southwest of Spain, specifically from the autonomous communities of Castilla La Mancha, Castilla León, and Extremadura.

Every woman in the clinical sample needed to fulfill the following inclusion criteria: being above 18 years old, having filed a police report against the purported abuser, meeting the prerequisites for enrollment in a center program, and possessing proficiency in the Spanish language. Exclusion criteria were comprised of intellectual disabilities hindering comprehension of the informed consent for study participation, psychiatric disorders (psychosis), substance dependencies (alcoholism and drug addiction), and instances where the individual was undergoing psychotherapeutic treatment.

The non-clinical sample needed to adhere to identical exclusion and inclusion criteria to the clinical sample. Additionally, having specific sociodemographic similarities with the chosen clinical sample was incorporated as an inclusion criterion.

Considering the backgrounds of the women in the complete sample, 47% originated from rural regions, contrasting with 53% from urban areas. The majority, constituting 87%, held Spanish nationality, with 7% being of African descent and 6% of Latin American descent.

The average age of the sampled population was 38.38 years (SD = 12.31). The participants typically initiated a romantic relationship with their partner at the age of 19.94 years and began cohabiting at 21.64 years. Concerning the number of children within the total sample, the average count was 2 (X = 1.91, SD = 1.12). Regarding religious affiliations in the overall sample, 88% identified as Catholic, 8% had no religious affiliation, and 4% adhered to religions other than Catholicism.

In relation to the existence of disability or handicap in the sample, 85% did not have one compared to 15% who did have handicap or disability, with an average degree of disability of 58.57% of the affected sample.

In the total sample, 52% reported having no siblings, while 21% had two siblings, 14% had four, and 13% had three siblings. Regarding the presence of living parents, 82% stated that one of their parents was alive, while 18% reported that both parents were deceased.

In the overall sample, 52% had completed primary education, 23% had secondary education, another 23% had university degrees, and 2% had no formal education. The marital status of the sample was comprised of 45% without any legal relationship, compared to 44% married and 11% in separation proceedings.

Regarding the occupations of the total sample, 31% were employed as homemakers, 16% were in the service sector, and 53% primarily worked as trade assistants. Concerning their employment status, 44% were actively employed, 45% were either unemployed or served as unpaid homemakers, and 11% were in other circumstances such as being on sick leave or retired.

In terms of socioeconomic status, it is notable that 6% were classified at a high level, 25% at a medium level, and 69% at a low level. With regards to an economic dependence, 54% of the total sample relied on financial support, with 44% dependent on their partners, 6% on the state, and 4% on their families, in contrast to 46% who were financially independent.

A comprehensive personal interview protocol, comprising 376 fields, was administered to each participant in both the clinical and non-clinical samples. This protocol aimed to gather thorough and structured information pertaining to various personal variables, including personal, socioeconomic, social/family details, social contexts, habits, motivations, and social participation.

The interview protocol was fashioned using established models utilized in police and judicial domains, such as the “Protection Order Request Form” (Formulario de Solicitud de Orden de Protección) issued by the Ministry of the Interior of Spain. This form is grounded in an action protocol established by the Security Forces and Coordination with Judicial Bodies for safeguarding victims of domestic and gender violence. Additionally, insights from semi-structured interviews conducted with victims of gender violence (Echeburúa et al., 1995) and data gathered from women exhibiting symptoms linked to intimate partner violence (IPV) informed the development of the protocol.

To assess the efficacy of the developed Stroop task, we utilized the Post-traumatic Stress Disorder Symptom Severity Scale (EGS), developed by Echeburúa, Corral, Amor, Zubizarreta, and Sarausa in 1995 (Echeburúa et al., 1995). The EGS is a hetero-evaluation scale designed as a structured interview aimed at evaluating the symptoms and intensity of post-traumatic stress disorder (PTSD) in individuals exposed to various traumatic events and sensitive to therapeutic interventions, following the diagnostic criteria outlined in the DSM-V. It comprises three subscales: reexperiencing, avoidance, and hyperarousal, totaling 17 items. Of these, 5 items pertain to reexperiencing symptoms, 7 to avoidance symptoms, and 5 to hyperarousal symptoms. The scale ranges from 0 to 51 on the global scale, from 0 to 15 on the reexperiencing subscale, from 0 to 21 on the avoidance subscale, and from 0 to 15 on the hyperarousal subscale. Responses are structured in a Likert-type format ranging from 0 to 3, based on the frequency and intensity of the symptoms.

Upon securing formal approval from the ethics committee to engage with patients, we proceeded with data collection and the creation of both the Emotional Stroop task for Detecting Women Victims of Domestic Violence and the Symptom Severity Scale for PTSD (EGS). This process involved conducting personal interviews, ensuring adherence to ethical principles including anonymity and confidentiality.

Prior to administering the instrument set, a quick assessment was conducted to screen for any color perception deficiencies (e.g., achromatism or dichromatism). This involved presenting colored squares (yellow, green, blue, and red) and asking participants to identify the corresponding colors. Following this, participants were provided with instructions and guidance to ensure proper understanding and execution of the Emotional Stroop task. They were instructed to verbally name the color in which the word was printed as quickly as possible, disregarding its meaning, within a 45-s timeframe.

### 2.5. Phase 5—Data Analysis

Quantitative data were analyzed using IBM SPSS Statistics, version 27.0. Following data cleaning, descriptive statistics including frequencies, means, and standard deviations were computed. To assess differences between samples, *t*-tests and within-subject effects tests were conducted. Additionally, correlational and linear regression analyses were performed to examine relationships and predictive models.

## 3. Results

Upon conducting the Kolmogorov–Smirnov test, it was determined that all the variables under study exhibited a normal distribution. Concerning the administration of the Post-traumatic Stress Disorder Symptom Severity Scale (EGS) test, the non-clinical sample yielded notably lower scores compared to the participants in the clinical group, as outlined in Table 5. To complement the analysis of differences between groups, Cohen’s d was calculated to determine the effect size in comparisons using the Post-Traumatic Stress Disorder Symptom Severity Scale (EGS). According to the established criteria for interpreting effect sizes (Cohen, 1988), these values correspond to large or very large effects, indicating that the observed differences between groups are not only statistically significant but also clinically relevant.

For the Emotional Stroop Test aimed at detecting PTSD in women victims of domestic violence, a comparative study was conducted between the non-clinical and clinical samples across the three types of sheets featuring neutral, positive, and negative words, assessing the count of correctly identified words (refer to Table 6).

The data presented in Table 6 revealed significant discrepancies across all three conditions (*p* < 0.001). Non-clinical participants exhibited higher scores across all conditions.

Based on these data, a general linear model, specifically an ANOVA with repeated measures, was conducted to analyze differences within the clinical and non-clinical groups. Results for the clinical group indicated significance at *p* = 0.001 and F = 8.19 (refer to Table 7), with estimated marginal means revealing differences between positive and negative word cards at *p* = 0.004 (α = 0.05), between negative and neutral word cards at *p* = 0.001 (α = 0.05), and between positive and neutral word cards at *p* = 0.191 (α = 0.05) (refer to Table 8). These outcomes suggest significant distinctions among the neutral, positive, and negative word sheets of the Emotional Stroop Test adapted for PTSD in women subjected to domestic violence.

In contrast, for the non-clinical population, the analysis yielded a significance level of *p* = 0.583 and an F value of 0.542 (refer to Table 7), with estimated marginal means indicating no significant differences between the positive and negative word sheets at *p* = 0.578 (α = 0.05), between the negative word sheets and the neutral ones at *p* = 0.283 (α = 0.05), and between the positive word sheets and the neutral ones at *p* = 0.650 (α = 0.05) (refer to Table 8). Consequently, we concluded that there are no significant distinctions among the neutral, positive, and negative word sheets of the Emotional Stroop Test adapted for PTSD in women subjected to domestic violence.

Additionally, to control Type I error risk in the Emotional Stroop Test, the Bonferroni correction was applied to account for multiple comparisons (refer to Table 9). After this adjustment, it was confirmed that differences in the group of women victims of intimate partner violence remained significant when comparing negative and neutral words (adjusted *p* = 0.006) and negative and positive words (adjusted *p* = 0.024), suggesting greater cognitive interference in response to negative stimuli. However, comparisons in the non-clinical group were no longer significant after the correction, ruling out the possibility of false positives in that group. These findings further support the validity of the Stroop task as a sensitive tool for assessing cognitive interference in women with PTSD symptoms resulting from intimate partner violence.

Ultimately, a Pearson Correlation test was conducted between the scores acquired from the negative word sheet of the Emotional Stroop Test for detecting PTSD in women victims of domestic violence and the scores derived from the Post-Traumatic Stress Disorder Symptom Severity Scale (EGS) applied to the clinical population. The analysis revealed a correlation coefficient of −0.528, with a significance level of *p* = 0.000.

**Figure 4:**
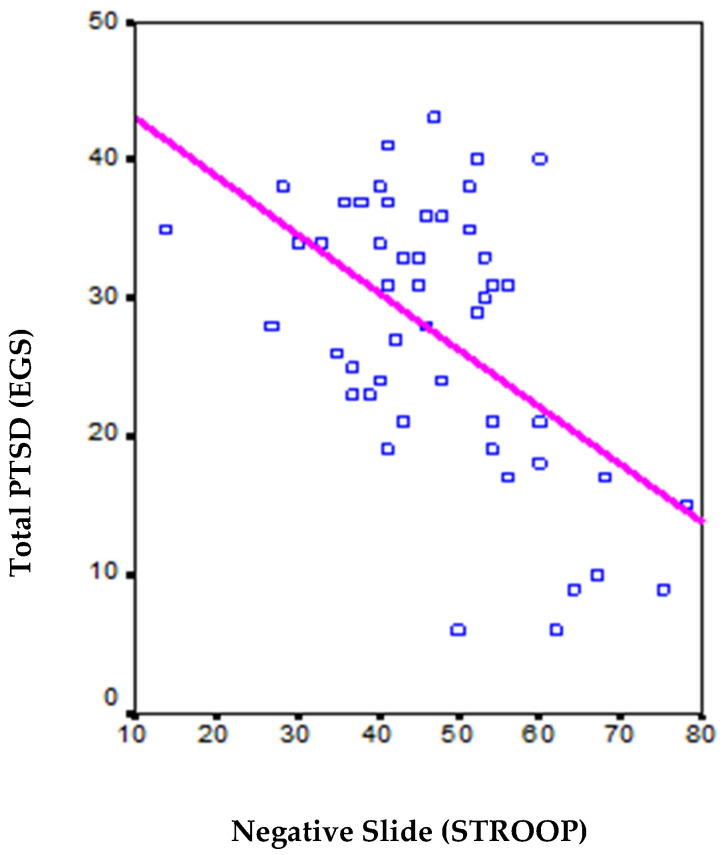
Graph of the inverse linear correlation between the scores obtained on the negative word sheet of the Emotional Stroop Test for Detection of PTSD in Women Victims of Domestic Violence and the scores obtained from the application of the Post-traumatic Stress Disorder Symptom Severity Scale (EGS), showing an R^2^ = 0.2787.

Therefore, it is evident that a notable inverse correlation is established in the test performance, indicating that low scores, reflecting fewer stimuli read on the negative word sheet (indicative of high hypervigilance), correspond to higher scores on the PTSD diagnostic test. This inverse correlation is visually depicted in Figure 4.

## 4. Discussion

The objective of this study was to create an Emotional Stroop Test tailored for identifying PTSD in Spanish-speaking women who are victims of domestic violence. The test aims to assess variations in emotional hypervigilance when exposed to stimuli associated with post-traumatic stress disorder resulting from domestic violence in comparison to the distinct levels indicated by the Post-traumatic Stress Disorder Symptom Severity Scale (EGS).

We hypothesize that individuals with elevated levels of PTSD, as assessed by the EGS, would demonstrate poorer performance in our version of the Stroop task designed for identifying PTSD in Spanish-speaking women victims of domestic violence.

To achieve this objective, the development of the test in Spanish encompassed three phases, followed by a validation phase in which the final questionnaire was administered to both a clinical sample (*n* = 50) and a non-clinical sample (*n* = 50). The clinical sample exhibited higher PTSD scores on the Post-traumatic Stress Disorder Symptom Severity Scale (EGS) test and lower correct response scores across all three sets of our Stroop task adaptation compared to the non-clinical sample. Moreover, there was a strong correlation between PTSD values obtained from the negative word sheet of our Stroop task adaption and the overall PTSD scores, all findings being statistically significant.

Considering that the Stroop task is utilized to illustrate attentional bias across various clinical populations, wherein participants typically exhibit prolonged reaction times when confronted with stimuli linked to their disorder or maladaptive behavior—specifically, they tend to identify the color of emotionally relevant words (such as those in the negative word sheet) more slowly than those with neutral emotional connotations (found in the sheet with neutral words)—our findings suggest that women victims of domestic violence (clinical sample) demonstrate greater interference (lower scores) on the negative word sheet, indicative of PTSD associated with domestic violence, compared to women in the non-clinical sample. This underscores the presence of attentional bias concerning domestic violence and its associated PTSD.

The outcomes align with prior research, such as the study by Sippel and Marshall (2011), which effectively adapted the Emotional Stroop task using words associated with shame alongside neutral terms to detect hypervigilance in situations involving self-shaming among women experiencing post-traumatic stress disorder due to domestic violence.

Our findings support earlier research indicating the significance of the Emotional Stroop task in PTSD assessment (Joyal et al., 2019; Yetter et al., 2023; Swick et al., 2024), highlighting its potential to address limitations associated with conventional tools, particularly in the context of women victims of domestic violence (Costello & Greenwald, 2022; Fang & Donley, 2022; García et al., 2023; Tolchin et al., 2023). This task reduced application time and expanded the range of environments in which it can be utilized due to its simplicity.

While our adaptation of the Stroop task for detecting PTSD in Spanish-speaking women victims of domestic violence has limitations, such as its inapplicability to individuals with color identification challenges, linguistic complexities, or cognitive impairments, considering the worldwide prevalence of PTSD linked to domestic violence (Pill et al., 2017) and the widespread use of Spanish (Olábarri Azagra, 2022), our research stands as a significant contribution to the global efforts in detecting and diagnosing PTSD associated with IPV, where the multiplicity of methodologies and their different efficacies (Maercker, 2021) hinder the development of psychobiological and neuroimaging tests (Neria, 2021; Dell’Oste et al., 2023; Jia et al., 2024) that could unequivocally corroborate the diagnosis of PTSD.

## 5. Patents

This work is in the patent formalization process.

## Figures and Tables

**Table 1:** Negative (or activating) words from the literature review and field work.

Negative Words from Literature Review			Negative Words from Field Work	
**Rape**	**Raped**	**Force**	Imbecile	**Death**
**Assault**	Incest	**Cry**	Torture	**Drunk**
Harasser	Vexed	Harassing	**Indefence**	**Disgraced**
Screaming	**Abused**	Male	**Pounding**	**Bitch**
Forcejacking	Penis	Flash	**Humiliation**	**Fool**
Trapped	**Victim**	Pain	Night	Door
Penetration	Shame	Engage	**Useless**	**Jealous**
Nightmare	**Bullier**	Disturb	Isolated	Denigrate
Attack	Blowjob	Exploit	**Children**	**Shame**
Runaway	Erection	Damage	**Threat**	**Whore**
		**Fear**		

Note: Words belonging to the negative categories that are integrated into the corresponding slide are highlighted in black.

**Table 2:** Negative (or activating) words proposed by the judges.

Negative Words Proposed by the Judges Sorted by Decreasing Score					
**Guilt**	**Alone**	Police	Bruises	Slave	Stupid
**Crazy**	**Thump**	Freedom	Pinches	Serve	Silly
**Knife**	**Fuck**	Illness	Bullshit	Drugs	Frustration
**Solitude**	**Bang**	Friend	Punching	Maid	Parents
**Isolated**	**Slap**	Hiding	Fighting	Lawyer	Shame
**Kicks**	Shotgun	Silence	Threats	Queens	Kill
**Warrior**	Tread	Dumb	Man	Sucking	Punish
**Dick**	Penalty	Punch	Screams	Shit	Report
**Beast**	Coup	Surprise	Vengeance	Servant	Marks

Note: Words belonging to the negative categories that are integrated into the corresponding slide are highlighted in black.

**Table 3:** Final selection of negative words distributed by semantic fields considering the previous selection by formal criteria.

Semantic Fields	Negative Words Selected According to Formal Criteria					
Physical violence	Thump	**Slap**	Bang	Kicks	Pounding	**Death**
Verbal violence	**Fool**	**Bitch**	Crazy			
Sexuality	Fuck	Dick	**Rape**			
Self-esteem	**Abused**	**Shame**	Alone			
Setting of domestic violence	Children	**Cry**	**Drunk**	**Knife**	Fear	Jealous

Note. Words belonging to the negative categories that are integrated into the corresponding slide are highlighted in black.

**Table 4:** Neutral, positive (or emotional), and negative (or activating) words.

Neutral Blade	Positive Blade (or Emotional)	Negative Blade (or Activating)
House	Fear	Slap
Stone	Suicide	Knife
Lift	Failure	Abused
Garden	Victim	Cry
Sofa	Disaster	Bitch
Curtain	Threat	Drunk
Cat	Terror	Rape
Heat	Fear	Shame
Flag	Accident	Death
Mat	Danger	Fool

**Table 5:** Results of the application of the Post-Traumatic Stress Disorder Symptom Severity Scale (EGS) test by non-clinical and clinical groups.

Variable	Group	*n*	*M* (SD)	t (df)	*p*	Cohen’s d
Total PTSD (EGS)	Non-Victims of IPV Women	50	6.78 (12.82)	9.08 (98)	0.000 *	1.815
	Women Victims of IPV	50	27.44 (9.73)			
Re-living PTSD (EGS)	Non-Victims of IPV Women	50	2.38 (4.70)	7.00 (98)	0.000 *	1.4
	Women Victims of IPV	50	8.92 (4.64)			
Avoidance PTSD (EGS)	Non-Victims of IPV Women	50	2.08 (4.49)	10.24 (98)	0.000 *	2.046
	Women Victims of IPV	50	10.58 (3.79)			
Psychophysiological activation PTSD (EGS)	Non-Victims of IPV Women	50	2.32 (4.67)	6.42 (98)	0.000 *	1.284
	Women Victims of IPV	50	7.94 (4.06)			

Note. *M*: mean; SD: standard deviation; *p*: *p*-value. *: significant for α = 0.05. Intimate partner violence (IPV); post-traumatic stress disorder (PTSD); Post-Traumatic Stress Disorder Symptom Severity Scale (EGS).

**Table 6:** Results for neutral, positive, and negative words scores by non-clinical and clinical groups.

Variable	Group	*n*	*M* (SD)
Neutral words	Non-Victims of IPV Women	50	58.34 (11.80)
	Women Victims of IPV	50	52.04 (11.10)
Positive (or emotional) words	Non-Victims of IPV Women	50	57.84 (15.58)
	Women Victims of IPV	50	50.48 (11.77)
Negative (or activating) words	Non-Victims of IPV Women	50	52.22 (14.97)
	Women Victims of IPV	50	47.28 (12.29)

Note. *M*: mean; SD: standard deviation; Intimate partner violence (IPV).

**Table 7:** Repeated-measures ANOVA for Emotional Stroop Test for screening of Post-traumatic Stress Disorder (PTSD) application, testing the within-subjects of groups. Greenhouse–Geisser statistics are used.

Group	*SS*	*Dƒ*	*MS*	*F*	*p*
Non-Victims of IPV Women	31.48	1.99	15.85	0.54	0.58
Women Victims of IPV	588.85	1.85	317.62	8.19	0.001 *
Error (Non-Victims of IPV Women)	2846.52	97.30	29.26		
Error (Women Victims of IPV)	3521.81	90.85	38.77		

Note. *SS*: sum of squares; *Df*: degrees of freedom; *MS*: mean sum; *F*: ANOVA coefficient; *p*: *p*-value. *: significant for α = 0.05. Intimate Partner Violence (IPV).

**Table 8:** Pairwise comparison for different types of slides of Emotional Stroop Test for screening of Post-traumatic Stress Disorder (PTSD) application results. Based on estimated marginal means.

Group	Stroop (I)	Stroop (J)	Mean Difference (I-J)	Std. Error	Sig.b	95% CI for Difference Lower Bound	95% CI for Difference Upper Bound
Non-Victims of IPV Women	1	2	0.50	1.09	0.650	−1.70	2.70
		3	1.12	1.03	0.283	−0.95	3.19
	2	1	−0.50	1.09	0.650	−2.70	1.70
		3	0.62	1.11	0.578	−1.60	2.84
	3	1	−1.12	1.03	0.283	−3.19	0.95
		2	−0.62	1.11	0.578	−2.84	1.60
Women Victims of IPV	1	2	1.56	1.18	0.191 *	−0.81	3.93
		3	4.76	1.35	0.001 *	2.06	7.46
	2	1	−1.56	1.18	0.191 *	−3.93	0.81
		3	3.20	1.06	0.004 *	1.08	5.32
	3	1	−4.76	1.35	0.001 *	−7.46	−2.06
		2	−3.20	1.06	0.004 *	−5.32	−1.08

Note. Stroop: Slides of Emotional Stroop Test for screening of Post-traumatic Stress Disorder (PTSD); 1: Slide of Neutral Words; 2: Slide of Positive Words; 3: Slide of Negative Words; *: significant for α = 0.05. Intimate Partner Violence (IPV).

**Table 9:** Bonferroni’s correction for multiple comparison in Emotional Stroop Test for screening of Post-traumatic Stress Disorder (PTSD) application results.

Comparison	Initial *p*-Value	Bonferroni *p*-Value
2 vs. 3 (Non-Victims)	0.283	1.0
3 vs. 1 (Non-Victims	0.283	1.0
1 vs. 2 (Victims)	0.191	1.0
2 vs. 3 (Victims)	0.004	0.024
3 vs. 1 (Victims)	0.001	0.006

Note. Comparison: Comparison between different slides of Emotional Stroop Test for screening for Post Traumatic Stress Disorder (PTSD); 1: Slide of Neutral Words; 2: Slide of Positive Words; 3: Slide of Negative Words; vs: versus.

## Data Availability

Data available upon request due to ethical and privacy restrictions.
